# Trends and Inequalities of Co-Occurring Obesity and Elevated Blood Pressure Among Chinese Children and Adolescents Aged 7–18 Years from 1985 to 2019 and Projections to 2030

**DOI:** 10.3390/nu17172828

**Published:** 2025-08-30

**Authors:** Tianyu Huang, Jiajia Dang, Jiaxin Li, Shan Cai, Yunfei Liu, Ziyue Chen, Yihang Zhang, Ruolan Yang, Peijin Hu, Jun Ma, Yi Song

**Affiliations:** Institute of Child and Adolescent Health, School of Public Health, Peking University, Beijing 100191, China; tyhuang@pku.edu.cn (T.H.);

**Keywords:** overweight/obesity, elevated blood pressure, child

## Abstract

**Background**: The co-occurrence of obesity and elevated blood pressure (EBP) in childhood represents a critical but underrecognized public health concern, with potential long-term consequences for cardiometabolic health. Understanding its trends and disparities is essential for early prevention strategies. **Methods**: This study analyzed data from 1,692,660 Han Chinese children and adolescents aged 7–18 years collected across seven waves of the Chinese National Survey on Students’ Constitution and Health (CNSSCH) from 1985 to 2019. Joinpoint regression was used to estimate temporal trends, and logistic generalized additive models were fitted to predict prevalence through 2030. **Results**: The prevalence of co-occurring obesity and EBP increased from 0.06% in 1985 to 2.36% in 2019 and is projected to reach 5.87% by 2030. A slowdown in the growth rate was observed approximately in 2000. Notably, rural areas experienced a faster and more recent rise, especially among girls, suggesting widening disparities. **Conclusions**: The growing dual burden of obesity and EBP in Chinese youth, especially in rural areas, calls for urgent and integrated interventions. Public health efforts must prioritize early prevention, with equitable policies that engage schools, families, and communities, particularly in underserved populations.

## 1. Introduction

As rapid socioeconomic transformation has occurred, the prevalence of obesity and high blood pressure (HBP) among Chinese children has risen sharply in recent decades, resulting in long-lasting adverse health consequences [1,2]. Evidence has revealed that obesity and HBP could progress into adulthood, serving as risk factors for cardiovascular problems later in life [3,4]. These issues not only jeopardize individuals’ physical and mental health throughout the course of their life, but also impose substantial healthcare and economic burdens at the national level [5].

Obesity has been identified as a key risk factor for HBP, and the co-occurrence of obesity and HBP during childhood and adolescence may lead to serious public health consequences across the course of life [6,7]. An analysis of National Health and Nutrition Examination Series (NHANES) data from 2015 to 2018 revealed that children with obesity were more likely to have hypertension than children with normal weight [8]. More critically, obesity and HBP share multiple modifiable risk factors, including dietary patterns, physical activity, sleep behaviors, and environmental exposures [9,10]. This convergence holds particular significance for preventive strategies, as targeted interventions addressing these factors could simultaneously mitigate both childhood obesity and hypertension, two major non-communicable diseases [5]. Early attention to the comorbidity of these conditions, with a focus on shared risk factors and high-risk populations, will efficiently improve the cardiometabolic health of the population and reduce their associated health–economic burdens.

Several international and Chinese studies have examined the impact of BMI on adolescent blood pressure [11,12]. For example, in a cohort of more than 63,000 overweight or obese children and adolescents from Europe, 35.3% had elevated blood pressure (EBP, defined as blood pressure above recommended thresholds on the basis of a single visit rather than repeated measurements), whereas only 6.1% of 14,298 normal-weight peers had EBP [13]. However, in young populations, existing studies are relatively dated and have rarely reported the prevalence of the co-occurrence of obesity and EBP in large samples, instead predominantly examining obesity as a risk factor for EBP, especially in the past two decades, when the nutritional environment across China has experienced profound changes. Understanding the current prevalence and secular trends of this comorbidity is essential for identifying high-risk groups, informing early prevention strategies, and guiding public health policy to mitigate the growing cardiovascular burden in children and adolescents.

Therefore, this study aimed to examine the secular trends of the co-occurrence of obesity and EBP among Han Chinese children and adolescents aged 7–18 years, with a projection of the prevalence of this co-occurrence by the year 2030. To capture a broader high-risk population, we also explored the trends in the co-occurrence of overweight/obesity (OWOB) and EBP. We sought to describe the evolving patterns and identify specific target populations for future intervention strategies.

## 2. Materials and Methods

The data were sourced from seven cross-sectional waves (1985, 1995, 2000, 2005, 2010, 2014, and 2019) of the Chinese National Survey on Students’ Constitution and Health (CNSSCH), a nationally representative survey of school-aged children via stratified cluster sampling. In each province, cities were categorized into three socioeconomic strata (upper, moderate, and low) on the basis of regional gross domestic product, total yearly income per capita, average food consumption per capita, the natural growth rate of the population, and the regional social welfare index. One city from each stratum was randomly selected. Within each selected city, a multistage sampling approach was used: middle and high schools were randomly selected on the basis of standardized procedures, followed by random selection of classes within each grade level for student participation in the survey. Informed consent was obtained from the participants and their guardians. The place of residence (urban/rural) for each participant was defined according to their *hukou* information.

A total of 1,692,660 Han Chinese children and adolescents aged 7–18 years were included in this study from 1985 to 2019 after 0.7% of the cases were removed because of missing values. The project was approved by the Medical Research Ethics Committee of the Peking University Health Science Centre (IRB00001052-19095).

Participants in the seven CNSSCH surveys underwent a complete anthropometric evaluation according to a standardized protocol, and evaluations were administered by trained personnel. All the measurements were conducted via standardized instruments, which were calibrated prior to use. Height and weight were measured with a portable wall-mounted stadiometer and standardized scale, and the mean values of three measurements were used. The measurement errors were controlled such that the height deviations did not exceed 0.5 cm and the weight deviations did not exceed 0.1 kg. Body mass index (BMI) was calculated as body weight (kg) divided by height (m) squared (kg/m^2^). Different BMI groups, including thinness, normal, overweight, and obese, were defined following the national health industry standard of screening for overweight and obesity among school-age children and adolescents (WS/T 586-2018) [14]. Blood pressure (BP) was measured with an auscultation mercury sphygmomanometer. Before measurement, the children were asked to sit for at least 10 min. Systolic BP and diastolic BP were calculated as the average of three measurements at a single visit for each child. An EBP was defined as a systolic or/and diastolic BP ≥ in the reference age-, sex-, and height-specific 95th centile, respectively, according to the reference for screening for elevated blood pressure among children and adolescents aged 7–18 years (WS/T 610-2018) [15].

Statistical analyses were performed via SPSS (version 27.0, IBM Corp., Armonk, NY, USA) and R (version 4.4.1, R Core Team, Vienna, Austria). Descriptive statistics were calculated for all variables, and Mantel-Haenszel chi-square tests were used to assess linear trends in prevalence from 1985 to 2019. For spatial visualization of urban-rural disparities, heatmaps were generated in R via the sf and ggplot2 packages. The urban-to-rural prevalence ratios were stratified by province and sex, with color gradients scaled to represent ratio ranges (<1 indicating rural predominance, >1 indicating urban predominance). For time trend analyses, we employed joinpoint regression (JoinPoint version 5.4.0; National Cancer Institute) to detect inflection points in crude prevalence. The annual percent change (APC) and average annual percent change (AAPC) were calculated as key outcome measures. On the basis of identified joinpoints, logistic generalized additive models (GAMs) with population weights were fitted to each subgroup, accommodating nonlinear trends and projecting the 2030 prevalence with 95% confidence intervals.

## 3. Results

### 3.1. Characteristics of the Participants and Distributions of Overweight/Obesity and Elevated Blood Pressure

The distribution of participants across sex, age groups, and urban/rural areas was relatively balanced in each survey wave. Over the study period, the prevalence of OWOB increased substantially from 0.69% in 1985 to 20.45% in 2019; the prevalence of obesity alone rose from 0.03% to 5.03%, corresponding to an increase of more than 150fold. In parallel, the prevalence of co-occurring OWOB and EBP increased markedly from 0.46% to 4.64%, and the prevalence of co-occurring obesity and EBP also surged from 0.06% in 1985 to 2.36% in 2019, reflecting a nearly 39-fold increase (Table 1).

### 3.2. Trends in the Prevalence of Co-Occurring Overweight/Obesity and Elevated Blood Pressure from 1985 to 2019

**Figure 1:**
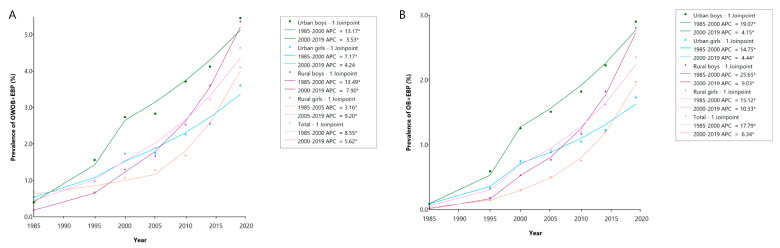
Joinpoint regression analysis of the prevalence of co-occurring OWOB and EBP (**A**) and co-occurring obesity and EBP (**B**) among Han children and adolescents aged 7–18 years in China from 1985 to 2019 stratified by urban/rural area and sex. Note: * indicates that the APC is significantly different from zero at the significance threshold of a *p* value < 0.05. Abbreviations: OWOB + EBP = co-occurring overweight/obesity and elevated blood pressure; OB + EBP = co-occurring obesity and elevated blood pressure; APC = annual percentage change.

The prevalence of co-occurring OWOB and EBP increased consistently across all subgroups from 1985 to 2019 (Figure 1A). Overall, the AAPC for the prevalence of co-occurring OWOB and EBP was 6.90% [95% confidence interval (CI): 5.86, 7.87], with the APC decreasing from 8.55% (95% CI: 6.50, 11.10) during the 1985–2000 period to 5.62% (95% CI: 1.52, 7.43) after an inflection point in 2000. Among urban boys, the AAPC was 7.68% (95% CI: 6.89, 8.56), with a decelerated increase after 2000. Urban girls had an AAPC of 5.52% (95% CI: 4.37, 6.59), also showing a slower upward trend during the post-2000 period. Rural boys experienced a more pronounced increase, with an AAPC of 10.33% (95% CI: 9.97, 10.70), and the growth rate slowed similarly after 2000. In contrast, rural girls presented an AAPC of 5.60% (95% CI: 4.48, 6.84), and the growth rate rose from the lowest (APC = 3.16, 95% CI: 0.50, 5.35) to the highest (APC = 9.20, 95% CI: 6.19, 14.47) in the four groups after the inflection point in 2005.

The prevalence of co-occurring obesity and EBP also exhibited consistent growth across all subgroups from 1985 to 2019 (Figure 1B). Overall, the AAPC for the prevalence of co-occurring obesity and EBP was 11.25% (95% CI: 10.45, 12.22), with the APC decreasing from 17.79% (95% CI: 14.86, 20.36) before 2000 to 6.34% (95% CI: 4.64, 7.58) from 2000 to 2019. Among urban boys, the AAPC was 10.49% (95% CI: 9.95, 11.02); urban girls had an AAPC of 8.87% (95% CI: 7.94, 9.97). Rural boys experienced a more pronounced increase, with an AAPC of 16.07% (95% CI: 15.42, 16.91), and rural girls showed an AAPC of 12.42% (95% CI: 12.16, 12.71). Notably, the growth rate of prevalence slowed, with the same inflection point occurring in 2000 across all four groups.

### 3.3. Inequalities in the Prevalence of Co-Occurring Overweight/Obesity and Elevated Blood Pressure from 1985 to 2019

**Figure 2:**
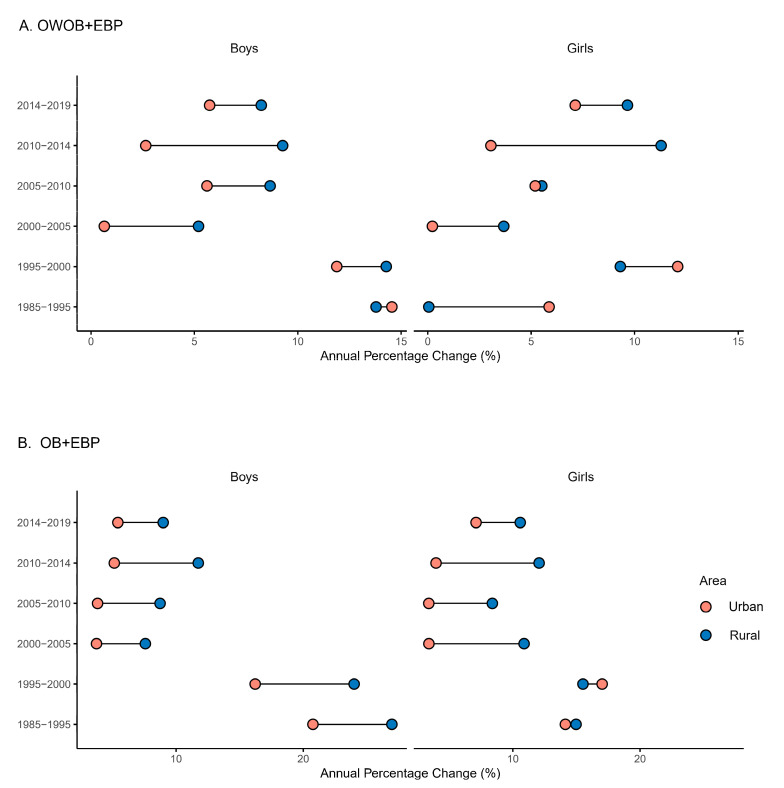
Inequalities in the APCs of the prevalence of co-occurring OWOB and EBP (**A**) and obesity and EBP (**B**) across different sexes and areas. Abbreviations: OWOB + EBP = co-occurring overweight/obesity and elevated blood pressure; OB + EBP = co-occurring obesity and elevated blood pressure.

**Figure 3:**
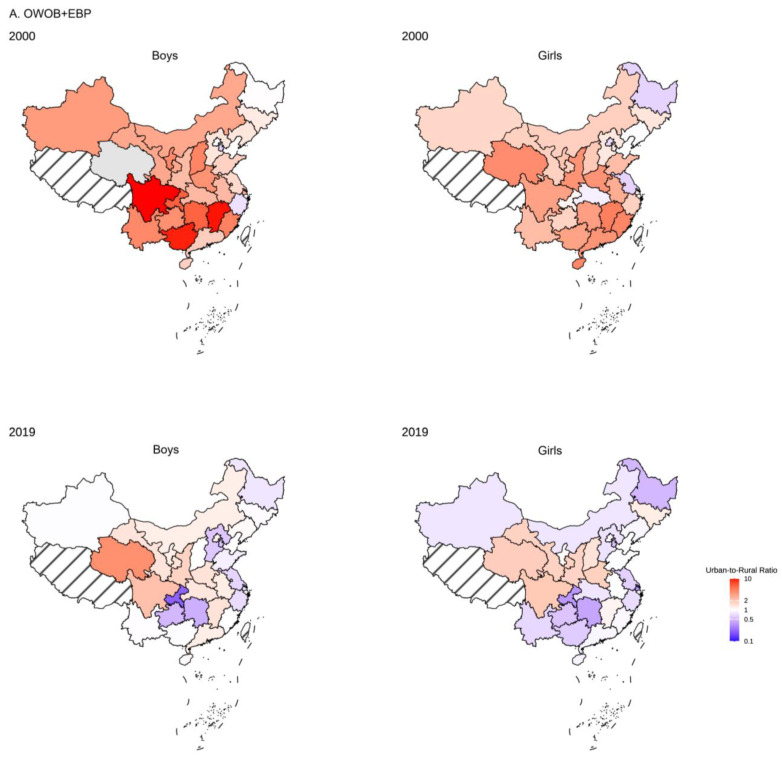

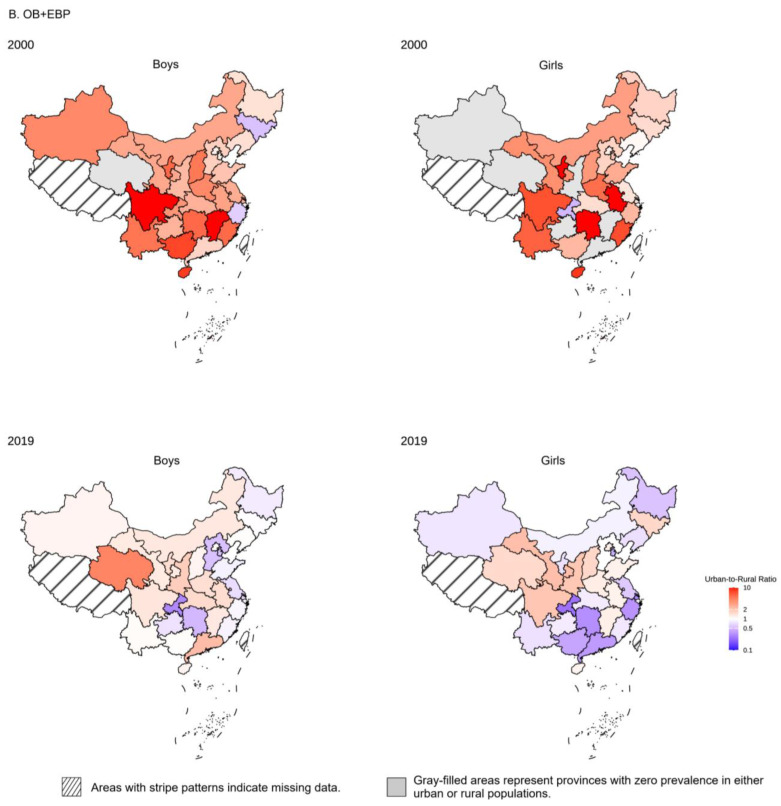
Regional distributions of the urban-to-rural ratios of co-occurring OWOB and EBP (**A**) and obesity and EBP (**B**) across different sexes in 2000 and 2019. Abbreviations: OWOB + EBP = co-occurring overweight/obesity and elevated blood pressure; OB + EBP = co-occurring obesity and elevated blood pressure.

Figure 2A shows the urban-rural inequalities in APCs of co-occurring OWOB and EBP from 1985 to 2019. From 1985 to 1995, urban boys presented a greater prevalence of APC than did their rural counterparts (urban-rural: 0.77%). However, this pattern reversed after 1995, with rural boys consistently demonstrating higher APCs thereafter. After 2000, the APC declined markedly for both urban and rural boys, while the urban–rural disparity in growth rates became more pronounced than it did in the pre-2000 period, with the peak level of inequality observed from 2010 to 2014 (urban-rural: −6.62%). During the periods of 1985–1995 and 1995–2000, urban girls presented greater APCs than rural girls did. However, this pattern reversed after 2000, with rural girls exhibiting consistently higher APCs thereafter. Unlike the trends observed in boys, the urban-rural disparity among girls demonstrated greater fluctuations over time. Notably, the peak level of inequality occurred during 2010–2014 (urban-rural: −8.23%), marking the period with the most pronounced difference in growth rates between urban and rural populations. Figure 3A shows the national geographical distribution of urban-to-rural prevalence ratios for co-occurring OWOB and EBP in 2000 and 2019. The analysis reveals evolving regional patterns during the period. While most provinces presented an urban predominance for both sexes in 2000, by 2019, eastern provinces predominantly presented a higher prevalence in rural areas. The central and western provinces maintained urban predominance throughout this period. Notably, the proportion of provinces with urban-to-rural ratios less than 1 (rural > urban) among both boys and girls increased significantly from 2000 to 2019. Although both sexes followed similar temporal trends, boys consistently exhibited stronger urban predominance than girls did in both 2000 and 2019.

Figure 2B shows the urban-rural inequalities in the APC of co-occurring obesity and EBP from 1985 to 2019. During the whole period, boys consistently demonstrated faster prevalence growth rates in rural areas than in urban areas. However, the year 2000 marked a sharp decline in APCs for both rural and urban male populations. The maximum urban-rural inequality in the APCs occurred from 1995 to 2000 (urban-rural: −7.78%), with the gap subsequently narrowing in the post-2000 period. Among girls, the urban-rural disparity in the prevalence of APC followed a different pattern. The pre-2000 period showed minimal differences between urban and rural populations, although urban girls presented slightly greater APCs from 1995 to 2000. After 2000, rural girls demonstrated consistently greater APCs, with peak disparities observed from 2010 to 2014 (urban-rural: −8.10%). Figure 3B shows the national geographical distribution of urban-to-rural prevalence ratios for co-occurring obesity and EBP in 2000 and 2019. Like co-occurring OWOB and EBP, while most provinces showed urban predominance for both sexes in 2000, eastern provinces predominantly presented a higher prevalence in rural areas by 2019. The central and western provinces maintained urban predominance throughout this period. The proportion of provinces with urban-to-rural ratios less than 1 (rural > urban) among both boys and girls increased significantly from 2000 to 2019. Although both sexes followed similar temporal trends, boys consistently exhibited stronger urban predominance than girls did in both 2000 and 2019.

### 3.4. Projections of the Prevalence of Co-Occurring Overweight/Obesity and Elevated Blood Pressure to 2030

**Figure 4:**
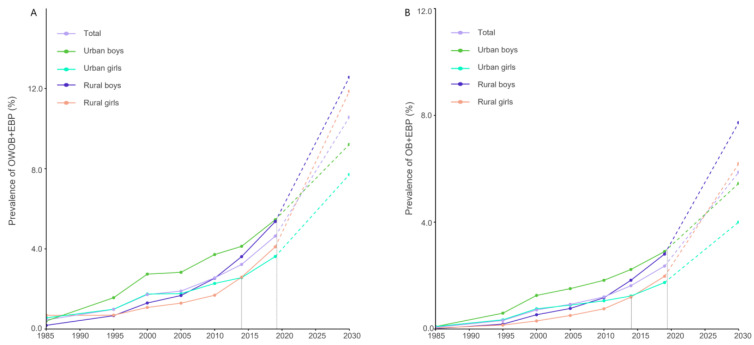
Prevalence from 1985 to 2019 and projection to 2030 of co-occurring OWOB and EBP (**A**) and co-occurring obesity and EBP (**B**) among Han children and adolescents aged 7–18 years in China, by urban/rural area and sex. Note: The dashed lines represent that the trends from 2019 to 2030 are based on forecasted data; the grey lines mark the time points of rural-urban crossovers. Abbreviations: OWOB + EBP = co-occurring overweight/obesity and elevated blood pressure; OB + EBP = co-occurring obesity and elevated blood pressure.

Figure 4 shows the trends in the prevalence of co-occurring OWOB/obesity and EBP, along with projections to 2030 among different sexes and areas. From 1985 to 2019, the prevalence of co-occurring OWOB and EBP increased significantly among urban boys (1985: 0.40%, 2019: 5.46%), urban girls (1985: 0.56%, 2019: 3.62%), rural boys (1985: 0.18%, 2019: 5.37%), and rural girls (1985: 0.69%, 2019: 4.10%). By 2030, these rates are projected to further increase to 9.22% (95% CI: 8.00, 10.44), 7.71% (95% CI: 6.35, 9.07), 12.57% (95% CI: 11.05, 14.09), and 11.87% (95% CI: 10.13, 13.60), and the overall prevalence is expected to be 10.58% in 2030. The prevalence of co-occurring obesity and EBP also significantly increased from 1985 to 2019. Among urban boys, urban girls, rural boys, and rural girls, the prevalence rates were 0.09%, 0.09%, 0.02%, and 0.04%, respectively, in 1985; increased to 2.90%, 1.74%, 2.81%, and 1.97%, respectively, in 2019; and increased to 5.45% (95% CI: 4.41, 6.49), 4.00% (95% CI: 2.96, 5.05), 7.73% (95% CI: 6.16, 9.29), and 6.19% (95% CI: 5.07, 7.31), respectively. The overall prevalence is expected to be 5.87% by 2030. Notably, in 2014, the prevalence of both co-occurring OWOB and EBP and co-occurring obesity and EBP became greater in rural girls than in urban girls, indicating a rural–urban crossover; among boys, this crossover is projected to have occurred after 2019.

## 4. Discussion

This study revealed a dramatic increase in the prevalence of co-occurring OWOB/obesity and EBP among Chinese youth from 1985 to 2019. While all the subgroups exhibited increasing trends, rural boys showed the steepest growth, followed by rural girls after 2005. A notable inflection point occurred in 2000, after which growth rates slowed but urban-rural disparities widened, peaking from 2010 to 2014. Eastern provinces shifted from urban to rural predominance from 2000 to 2019, whereas the central and western regions maintained urban predominance. Boys consistently demonstrated stronger urban predominance than girls did. Projections suggest potential alarming escalations by 2030, particularly in rural areas; a rural-urban prevalence crossover emerged among girls by 2014 and is anticipated among boys post-2019. These findings underscore an accelerating burden of co-occurring OWOB/obesity and EBP, with rural populations increasingly being disadvantaged.

These findings align with the literature, which indicate a pronounced and worsening co-occurring trend between obesity and EBP in children and adolescents. For example, a study conducted in China, India, and Mexico revealed that, compared with normal-weight children, overweight children had approximately 1.7–2.3 times greater odds of hypertension, whereas the risk among obese children was 3.5–5.5 times greater [16]. Despite these findings, large-scale studies reporting the prevalence of co-occurring obesity and EBP in children remain limited. Our study contributes to filling this gap by providing prevalence data on this co-occurrence in a sizable cohort. Moreover, while previous studies have often examined obesity as a risk factor for elevated blood pressure [13,16], our study emphasizes the importance of monitoring both conditions concurrently to better understand their relationship and potential combined impact on cardiovascular health in pediatric populations.

The co-occurrence of OWOB/obesity and EBP in childhood may have synergistic effects on cardiometabolic health throughout the course of life. They jointly accelerate endothelial dysfunction, insulin resistance, and left ventricular hypertrophy, significantly increasing the risk of early-onset cardiovascular disease and type 2 diabetes in adulthood [17]. The rapid increase in the co-occurrence of these risk factors in China reflects a broader shift in children’s health environments with social transitions, influenced by unhealthy lifestyles and exposure to obesogenic environments [5]. Interestingly, we observed a slowing growth rate approximately in 2000, which may correspond to the implementation of several national health and education policies during that period, emphasizing the substantial potential for policy interventions in mitigating obesity and EBP in children [18].

Marked urban–rural and sex disparities were observed in the evolving trends of co-occurring OWOB/obesity and EBP. Our analysis is consistent with previous obesity-focused studies [2,18], which revealed that nutritional transition affects not only obesity, but also the entire cardiometabolic system. As a result, co-occurring OWOB/obesity and EBP follow trends similar to those of obesity alone. Before 2000, rural growth rates surpassed those in urban areas, with many eastern provinces demonstrating complete rural-urban prevalence reversals by 2019. This shift highlights rural populations as a critical priority for intervention, which may be increasingly affected by obesogenic environments and inadequate preventive measures. Although no significant urban-rural reversal has yet been observed in some central and western regions, these areas have demonstrated alarming acceleration in rural prevalence rates that warrant urgent attention. While regions with higher socioeconomic status (SES) can effectively mitigate health risks through robust school health systems and substantial health education investments, rural areas in less developed provinces face systemic barriers in securing adequate health resource allocation.

Moreover, rural girls represent a doubly vulnerable group. The steep increase in their prevalence may reflect biological susceptibility, as evidenced by stronger BMI–blood pressure associations in females [19]. Current policies insufficiently address these intersecting vulnerabilities, as demonstrated by the persistent rural female health penalty despite nationwide economic growth. These findings demand targeted strategies, including prioritizing rural areas through the implementation of gender-responsive school-based programs [20].

Our projections indicate that, if the current trend continues, by 2030, the prevalence of co-occurring OWOB and EBP among Chinese children and adolescents is expected to be 10.58%, with an even greater burden anticipated in rural areas. In recent years, China has introduced major national initiatives such as the Healthy China 2030 strategy and the Weight Management Year campaign, aiming to curb the rising tide of childhood obesity and its related complications. However, targeted research and policy investment for rural children and adolescents remain insufficient. Given the multifactorial nature of obesity and hypertension, effective interventions require a multisectoral approach that engages families, schools, and communities to create a healthier social and natural environment, which could contribute to the mitigation of more than one health issue [5]. However, although our projections provide valuable insights into future trends, they should be interpreted with caution, as they are inherently dependent on assumptions and may be influenced by unpredictable external factors such as changes in health policies, socioeconomic development, or lifestyle shifts.

The findings of this study are subject to several limitations. First, the diagnosis of EBP was based on single-visit measurements rather than repeated assessments across multiple days, which may overestimate the prevalence. Although standardized protocols were followed, single-occasion measurements may overestimate or underestimate the true prevalence of elevated blood pressure due to situational and subjective factors. Consequently, the reported prevalence should be interpreted with caution. Second, our projections are model-based and may be influenced by future policy changes or unforeseen factors.

## 5. Conclusions

This study revealed a marked increase in the prevalence of co-occurring OWOB/obesity and EBP among Chinese children and adolescents from 1985 to 2019. The rise was most pronounced in rural boys and, after 2005, in rural girls. However, a notable slowdown was identified after 2000, accompanied by widening urban–rural disparities. Regional differences were also evident, with eastern provinces shifting to rural predominance, whereas the central and western regions remained predominantly urban. Projections suggest continued escalation by 2030, particularly in rural areas, with a rural–urban crossover already observed among girls and expected among boys after 2019. These findings highlight a growing burden of childhood cardiometabolic risk and call for attention to urban–rural and gender disparities. Policy interventions may play a critical role in modifying long-term trends. Our results underscore the urgent need for comprehensive and equitable strategies to prevent obesity and EBP in early life. Strengthening health promotion in underserved populations, especially rural girls, will be essential to achieving national goals for child health and noncommunicable disease prevention.

## Figures and Tables

**Table 1:** Characteristics of the participants included in each survey.

	1985 (*n* = 407,692)	1995 (*n* = 204,393)	2000 (*n* = 211,630)	2005 (*n* = 231,613)	2010 (*n* = 215,238)	2014 (*n* = 213,481)	2019 (*n* = 208,613)	χ^2^	*p* Value
Urban/rural area, *n* (%)								4.232	0.040
Urban	204,436 (50.14)	103,633 (50.70)	106,366 (50.26)	116,509 (50.30)	107,525 (49.96)	106,849 (50.05)	104,437 (50.06)		
Rural	203,256 (49.86)	100,760 (49.30)	105,264 (49.74)	115,104 (49.70)	107,713 (50.04)	106,632 (49.95)	104,176 (49.94)		
Sex, *n* (%)								0.021	0.886
Boys	203,887 (50.01)	102,804 (50.30)	105,888 (50.03)	116,437 (50.27)	107,627 (50.00)	106,774 (50.02)	104,518 (50.10)		
Girls	203,805 (49.99)	101,589 (49.70)	105,742 (49.97)	115,176 (49.73)	107,611 (50.00)	106,707 (49.98)	104,095 (49.90)		
Age groups, *n* (%)								98.192	<0.001
7–9 years	101,627 (24.93)	48,943 (23.95)	53,276 (25.17)	57,836 (24.97)	53,820 (25.00)	53,482 (25.05)	53,396 (25.60)		
10–12 years	102,245 (25.08)	52,130 (25.50)	53,556 (25.31)	58,360 (25.20)	53,895 (25.04)	53,666 (25.14)	53,524 (25.66)		
13–15 years	102,148 (25.06)	51,850 (25.37)	53,099 (25.09)	58,317 (25.18)	53,852 (25.02)	53,804 (25.20)	53,078 (25.44)		
16–18 years	101,672 (24.94)	51,470 (25.18)	51,699 (24.43)	57,100 (24.65)	53,671 (24.94)	52,529 (24.61)	48,615 (23.30)		
Overweight, *n* (%)	5529 (1.36)	8817 (4.31)	14,552 (6.88)	20020 (8.64)	21,637 (10.05)	26,820 (12.56)	29,462 (14.12)	48,569.924	<0.001
Obesity, *n* (%)	700 (0.17)	2986 (1.46)	6723 (3.18)	10,795 (4.66)	11,805 (5.48)	16,969 (7.95)	21,189 (10.16)	45,271.719	<0.001
EBP, *n* (%)	65,642 (16.10)	15,807 (7.73)	19,351 (9.14)	15,968 (6.89)	19,188 (8.91)	18,437 (8.64)	25,727 (12.33)	6366.311	<0.001
Co-occurring OWOB and EBP, *n* (%)	1865 (0.46)	2004 (0.98)	3637 (1.72)	4387 (1.89)	5495 (2.55)	6881 (3.22)	9675 (4.64)	14,200.016	<0.001
Co-occurring obesity and EBP, *n* (%)	240 (0.06)	652 (0.32)	1512 (0.71)	2139 (0.92)	2587 (1.20)	3460 (1.62)	4917 (2.36)	9555.582	<0.001

## Data Availability

The data presented in this study are available upon request from the corresponding author.
